# Involvement of persons with lived experience in mental health research: recommendations

**DOI:** 10.1016/j.nsa.2026.107009

**Published:** 2026-05-12

**Authors:** K. Kleine Schaars, Allan H. Young, Ramona Moldovan, Erik Van der Eycken, Falk Schuster, Jan Klopper, Urs Heilbronner, Martien J.H. Kas, The PSY-PGx Consortium, Kristian Kleine Schaars, Sanah Bedi, Annet Clerx, Janet Strozeski, Allan H. Young, Mario Juruena, Daniel Silman, Urs Heilbronner, Monika Budde, Alba Navarro-Flores, Magnus Ingelman-Sundberg, Marin Jukic, Martien J.H. Kas, Raj Jagesar, Andra Ciucă, Ramona Moldovan, Markus M. Nöthen, Per Hoffmann, Carina M. Mathey, Alexandra Philipsen, Laura L. Kilarski, Jonathan Laatsch, Tehila Cohen, Estee Rebibo, Noam Shomron, Erik Van der Eycken, Nigel Olisa, Eduard Vieta, Natalia E. Fares-Otero, Thomas G. Schulze, Therese van Amelsvoort, Bea Campforts, Emma de Brabander, Moritz J. Rossner, Sven P. Wichert, Roos van Westrhenen, R. van Westrhenen

**Affiliations:** m Outpatient Clinic Pharmacogenetics, Parnassia Psychiatric Institute, Amsterdam, the Netherlands; n Centre for Human Drug Research, Leiden, the Netherlands; o Leiden University Medical Center, Leiden, the Netherlands; p Institute of Psychiatry, Psychology and Neuroscience, King's College London, London, United Kingdom; q Division of Psychiatry, Imperial College London, United Kingdom; r Institute of Psychiatric Phenomics and Genomics (IPPG), LMU University Hospital, LMU Munich, Germany; s International Max Planck Research School for Translational Psychiatry (IMPRS-TP), Munich, Germany; t Department of Physiology & Pharmacology, Karolinska Institute, Sweden; u Faculty of Pharmacy, University of Belgrade, Serbia; v Groningen Institute for Evolutionary Life Sciences, Faculty of Science & Engineering, University of Groningen, the Netherlands; w Department of Psychology, Babeş-Bolyai University, Romania; x Division of Evolution and Genomic Sciences, University of Manchester, United Kingdom; y Manchester Centre for Genomic Medicine, Manchester University Hospitals NHS Foundation Trust, United Kingdom; z Institute of Human Genetics, University Hospital of Bonn, Germany; aa Department of Psychiatry and Psychotherapy, University of Bonn, Germany; ab Gray Faculty of Medical and Health Sciences, Tel Aviv University, Tel Aviv, Israel; ac Global Alliance of Mental Illness Advocacy Networks-Europe (GAMIAN-Europe), Belgium; ad Bipolar and Depressive Disorders Unit, Department of Psychiatry and Psychology, Hospital Clinic, Institute of Neurosciences (UBNeuro), IDIBAPS, CIBERSAM, University of Barcelona, Catalonia, Spain; ae Department of Psychiatry and Behavioral Sciences, Norton College of Medicine, SUNY Upstate Medical University, Syracuse, NY, USA; af World Psychiatric Association, Geneva, Switzerland; ag Maastricht University, Maastricht, the Netherlands; ah Systasy Bioscience GmbH, Germany; ai St. John's National Academy of Health Sciences, Bangalore, India; aj Institute of Psychiatry, Psychology & Neuroscience (IoPPN,) King's College London, London, United Kingdom; a Outpatient Clinic Pharmacogenetics, Parnassia Psychiatric Institute, Amsterdam, the Netherlands; b Centre for Human Drug Research, Leiden, the Netherlands; c Leiden University Medical Center, Leiden, the Netherlands; d Division of Psychiatry, Imperial College London, United Kingdom; e Institute of Psychiatry, Psychology and Neuroscience, King's College London, London, United Kingdom; f Division of Evolution and Genomic Sciences, School of Biological Science, University of Manchester, United Kingdom; g Manchester Centre for Genomic Medicine, St Mary's Hospital, Manchester University Hospitals NHS Foundation Trust, Manchester Academic Health Science Centre, Manchester, United Kingdom; h Department of Psychology, Babeş-Bolyai University, Romania; i Global Alliance of Mental Illness Advocacy Networks-Europe (GAMIAN-Europe), Brussels, Belgium; j Institute of Psychiatric Phenomics and Genomics (IPPG), LMU University Hospital, LMU Munich, Germany; k Groningen Institute for Evolutionary Life Sciences, University of Groningen, the Netherlands; l St. John's National Academy of Health Sciences, Bangalore, India

## Abstract

Although the benefits of involvement of persons with lived experience in mental health research have been widely demonstrated, consistent implementation remains challenging. During the 36th ECNP congress in Barcelona, a session was hosted, discussing the collaboration between clinicians, researchers and the European patient organisation GAMIAN-Europe within the PSY-PGx Project. This short communication presents key themes emerging from this session and presents recommendations to support involvement of persons with lived experience in mental health research. Within the PSY-PGx project, persons with lived experience were involved from early on, which has contributed to active patient participation throughout the project. The recommendations may guide future research projects in optimizing patient involvement.

## 1 Introduction

### 1.1 Involvement of persons with lived experience in mental health research

Patient & Public Involvement (PPI) in scientific research is well established and increasingly recognised as an important factor in high-quality research, participant communication, ethics procedures and dissemination (Domecq et al., 2014; Skovlund et al., 2020; Prebeg et al., 2023). Still, implementation of PPI remains inconsistent, and many research projects do not yet fully integrate the potential that persons with lived experience can bring to the research process. Clear guidance on how meaningful involvement can best be achieved are lacking (Domecq et al., 2014; Skovlund et al., 2020; Prebeg et al., 2023). A major development was introduced in 2013 with the revision of the Declaration of Helsinki, which pointed out that participants should always be given access to relevant study outcomes (Association, 2013). This reinforced the principle that research participation should include transparency and reciprocal communication. Meaningful PPI has several well documented benefits. It is described that PPI can increase study enrolment but also decrease drop-out (Edwards et al., 2011). Also, it has been demonstrated that collaboration with persons with lived experience in study development can result in new findings and can guide the development of more patient-focused endpoints (Wit et al., 2013). However, performative implementation risks reduce PPI to a procedural requirement rather than meaningful collaboration, and studies might run the risk of becoming tokenistic. Recently, PPI has become a topic of interest in the scientific community. A recent study protocol in mental health research highlights that meaningful involvement is still inconsistent and sets out to assess barriers and enablers (Anand-Vembar et al., 2025). A recent scoping review examined 20 review papers covering 429 studies on PPI in psychiatric research, showing that PPI is increasingly being used to improve research relevance and dissemination through the use of advisory groups, interviews and focus groups. However, the level of participation varied considerably. Key facilitators consisted of trust, clear communication and strong relationships, while the most frequently mentioned barrier was the power imbalance between participants and researchers (Werner et al., 2025). It is important that a sense of urgency of PPI is created to strive for the most optimal collaboration between researchers, clinicians, and participants.

### 1.2 Personal recovery as an outcome measure

Traditionally, the way study outcomes are captured often rely on clinician rated symptom measures and functional restoration (Keller, 2004; Macpherson et al., 2016). Although these aspects are valuable, they might not capture the true extent of recovery, which is a non-linear, multidimensional process. Personal recovery has therefore emerged as a complementary outcome of research, focusing on living a meaningful life despite psychiatric difficulties, emphasising the needs of patients and overcoming disease related limitations (Deegan, 1988). The CHIME framework (Connectedness, Hope, Identity, Meaning, and Empowerment dimensions) has become a widely used model to assess the different dimensions of personal recovery (Leamy et al., 2011). The Recovery Assessment Scale (RAS) is a questionnaire used to quantify the dimensions of the CHIME framework (Law et al., 2012). The RAS is a patient reported, pan-diagnostic outcome measure to be used in both clinical and research settings. A recent meta-analysis highlights the sensitivity of the RAS to change (Pelgrim et al., 2026). Focusing more on patient-centred outcomes in clinical studies, will increase the likelihood contributing to improvement of the well-being and quality of life of individuals with psychiatric disorders.

## 2 Methods

### 2.1 The ECNP patient session

During the 36th yearly congress of the European College of Neuropsychopharmacology (ECNP) in Barcelona, a session was hosted to discuss the collaboration between professionals (clinicians and researchers) and the European patient organisation GAMIAN-Europe within the PSY-PGx project, a non-commercial, worldwide, investigator-initiated Horizon2020 funded project on implementation of pharmacogenetics in psychiatry, www.PSY-PGx.org (Pelgrim et al., 2024; Heilbronner et al., 2025; van Westrhenen et al., 2021). Pharmacogenetic information can be used to adjust medication to reduce side effects and increase efficiency.

During the ECNP session, short presentations first highlighted the involvement in study design of persons with lived experience within the project. Next, a plenary panel discussion was held. Panel members reflected on their experience with mental health research. Clinicians and researchers in the audience were invited to share their experiences and questions regarding PPI. Notes were taken of all topics and questions of this interactive discussion which were to be assessed and categorised later.

### 2.2 Practical recommendations

The recommendations presented in this short communication were based upon the themes discussed during the session. Draft recommendations were formulated in collaboration with persons with lived experience, clinicians and researchers involved in the PSY-PGx project. The aim was to produce practical recommendations that are directly applicable in mental health research.

## 3 Key themes emerging from the patient session

The session highlighted three recurring themes relevant to patient involvement in mental health research: burden associated with study procedures, the relevance of patient centred study endpoints and the importance of trust between participants and researchers.

First, patients that had experience participating in clinical trials, described the potentially burdensome research procedures and psychiatric examinations, particularly for those with severe mental disorders who may experience difficulty in processing large amounts of information during a visit. Lengthy appointments, repeated measures and extensive questionnaires, were all described as mentally demanding and may discourage continued participation. Difficulties recalling information discussed during previous visits might also hinder effective communication and quality of data. Practical adjustments were suggested such as: designing questionnaires in shorter digital format, displaying or presenting one question at a time, improving accessibility and providing real time feedback on completion time. Additionally, enabling self-assessment from home or at individual appropriate moments could alleviate stress and improve feasibility for participants.

Second, the session emphasised the limitations of current outcome measures in psychiatric research. Many research projects assess remission through symptom based and clinician rated questionnaires. While these measures remain important and provide insight into disease progression, it fails to capture the broader experience of recovery. Participants highlighted the importance of outcome measures that capture personal recovery. Personal recovery has emerged as an outcome of interest in scientific research. The RAS could help satisfy the need for more patient-centred outcomes (Law et al., 2012; Pelgrim et al., 2026).

Lastly, trust between participant and researcher was discussed and emerged as central condition for meaningful study participation. In many cases, contact between participant and research staff is brief and fragmented, making it difficult to establish a meaningful bond of trust. Participants indicated that a meaningful bond of trust increases willingness to share sensitive experiences, to provide feedback, and to remain engaged during the project resulting in reduced study dropout rates. Most of the key themes identified during this session were also highlighted in the scoping review by Werner and colleagues (Werner et al., 2025), emphasising the relevance of these identified themes.

## 4 Examples from PSY-PGx

### 4.1 Involvement of GAMIAN-Europe and the Patient Advisory Board

**Fig. 1:**
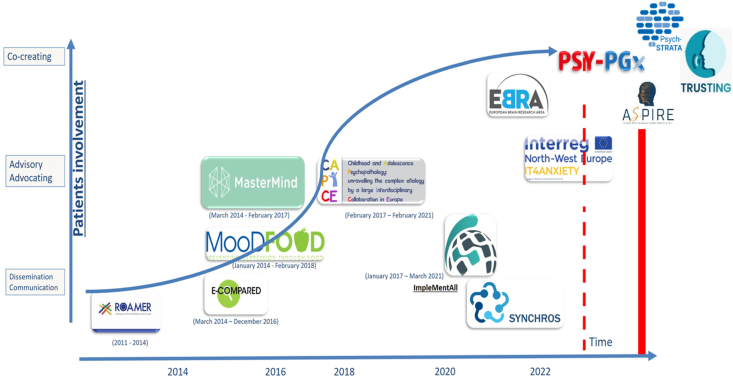
Level of patient involvement (PPI) in PSY-PGx as opposed to other studies in which GAMIAN-Europe has been involved.

The PSY-PGx project is a large investigator-initiated, non-commercial, worldwide project on implementation of pharmacogenetics in clinical psychiatry. One if the 8 work packages contains a randomised controlled trial (Pelgrim et al., 2024; van Westrhenen et al., 2025). Within this project, collaboration with persons with lived experience, was established through GAMIAN-Europe to ensure integrating the perspective of persons with lived experience throughout study development and execution. Furthermore, GAMIAN-Europe provides input during the trial and is asked to contribute to data collection and interpretation. GAMIAN's principal activities relate to advocacy, policy information, education, awareness-raising, addressing stigma and partnership building in EU funded research. All these aspects offer opportunities for growth, not only during a research project but also before and after. PSY-PGx has reached a high level of PPI together with GAMIAN as depicted in Fig. 1.

**Fig. 2:**
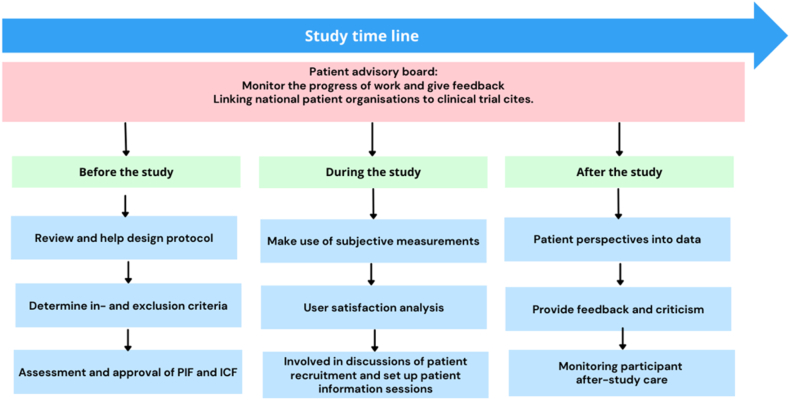
Visualization of patient involvement in the PSY-PGx project.

Prior to study initiation, GAMIAN-Europe was consulted by the Chief Principal Investigator on study design and participant-related procedures (see Fig. 2). Subsequently, a Patient Advisory Board (PAB) was established, consisting of individuals with lived experience from diverse European countries and mental health backgrounds. The PAB was tasked with offering insights into elements such as the clinical study protocol, dissemination material, and patient material such as consent forms. Moreover, the PAB facilitated connections with national patient organizations in The Netherlands, Belgium, Germany, Romania, and UK to establish contact with the local PSY-PGx clinical trial sites and awareness of pharmacogenetics in general.

Throughout the project, the PAB monitors its advancement, evaluates progress, and provides feedback to the consortium. Regular updates on project progress are shared with the PAB, which convenes regularly (e.g. coinciding with the consortium's general assembly).

GAMIAN-Europe also contributes expertise in participant recruitment strategies, defining patient input frameworks, and addressing cultural nuances. Given the project's global scope, cultural diversity presents challenges ranging from language barriers to societal norms. Patient organizations like GAMIAN offer valuable perspectives from individuals with varied cultural backgrounds, aiding in understanding and navigating these complexities.

### 4.2 Participant-centred procedures within PSY-PGx

Several patient-centred elements were integrated into the PSY-PGx study design. Interim measurements of participant satisfaction are used to assess participant involvement. Next, in collaboration with GAMIAN-Europe, a user satisfaction analysis of the BeHapp mobile application, utilized in clinical practice, was conducted to improve usability (Eskes et al., 2016; Jongs et al., 2020). In addition, the RAS was included as a primary outcome measure, which prioritizes patient-assessed subjective remission (Law et al., 2012; Corrigan et al., 2004). This approach provides insights into participants' perspectives on their mental health journey in a structured manner and reflects the project's aim to include outcomes that better align with the experience of participants. Also, stakeholder information sessions were organised to map all attitudes, ideas and potential gaps regarding implementation of pharmacogenetics in psychiatry as assessed from the participants in clinical trials. A non-commercial video was developed, in co-creation with GAMIAN-Europe, with 4 persons with lived experience explaining how pharmacogenetics helped them to find the right medication, which is available on YouTube (link) and published on the PSY-PGx YouTube channel.

### 4.3 Accessibility

The PSY-PGx consortium attempts to publish its findings in open-access journals. Open access ensures that research outcomes, generated with the voluntary participation of participants, remain accessible to all stakeholders, including persons with lived experience and caregivers, not solely restricted to healthcare professionals. This transparency fosters innovation, dissemination and motivates participants by acknowledging their contributions through openly available publications.

## 5 Recommendations

Based on themes discussed during the ECNP session, the following recommendations may support more meaningful PPI in mental health research.

1. Structured summaries after study visits
  o Participants may experience difficulty retaining information discussed during study visits, particularly due to extensive nature of assessments and complex explanations. Providing written summaries post-visit, along with a short overview of subsequent appointments, could significantly improve recall, support, adherence and reduce likelihood of missing follow-up data.
2. Use of participant reported outcome measures
  o Traditional clinician-rated questionnaires fail to capture the multidimensional nature of recovery in mental health disorders. Including questionnaires like the RAS, which focus on personal recovery, can help provide insight into autonomy, functioning and perceived recovery.
3. Reducing participant burden
  o Study visits should be adapted to minimise unnecessary burden of participants. Introducing options for home-based questionnaire completion or utilizing user-friendly formats such as video-guided or mobile phone questionnaires could alleviate this burden. Clear and appealing visual design of questionnaires with realistic time to complete estimations, might further improve patient engagement.
4. Communication of study outcomes
  o Clear communication of study outcomes is essential for fostering trust and transparency. Research findings should be communicated in a comprehensive manner and accessible language whenever possible. Persons with lived experience can play a pivotal role in translating complex scientific findings into understandable formats.
5. Evaluation of participant experience
  o Actively evaluating participant experience with the help of questionnaires and post-appointment discussions, should be used to gather insights into patients' experiences and identify areas for improvement. These may identify practical barriers that would otherwise remain unnoticed.
6. Involvement in interpretation and dissemination
  o Persons with lived experience may contribute meaningful interpretations of findings particularly regarding qualitative outcomes and participant experiences. Their involvement in the design and review of dissemination material may also improve their relevance, clarity, and accessibility.
7. Dealing with preconceptions:
  o Persons with lived experience can play a pivotal role in overcoming misconceptions about research, including concerns about medication or institutional motives. By leveraging their experience and expertise, they can help address such concerns by contributing credible perspectives during participant communication and outreach.

## 6 Conclusion

Although the benefit of PPI has been widely proven, consistent implementation in mental health research remains limited. Further progress can be made, particularly when it comes to improving participant relevant outcome measures and creating accessibly and less burdensome study procedures. This may increase incentive to participate in clinical studies and thereby enhance recruitment which remains a challenge across clinical trials. Within PSY-PGx, involvement of persons with lived experience from an early development stage has stimulated active participation throughout the project.

## Funding and declarations

The authors declare no competing interests for this work. This study was conducted as part of the PSY-PGx project funded in the context of European Union's Horizon 2020 research and innovation program under grant agreement No. 945151 (chief principal investigator: RvW). RvW has served/serves as an expert/received compensation from Lundbeck, Illumina, Benecke, PsyFar, Baxter, Chipsoft, NVvP, KNMP, KNMG, WPA, European union, UK Medical research Council, University Oslo, University Utrecht, Health Base.

EV has received grants and served as consultant, advisor or CME speaker for the following entities: AB-Biotics, Abbott, AbbVie, Adamed, Adium, Alcediag, Angelini, Biogen, Beckley-Psytech, Biohaven, Boehringer-Ingelheim, Casen-Recordati, Celon Pharma, Compass, Dainippon Sumitomo Pharma, Esteve, Ethypharm, Ferrer, Gedeon Richter, GH Research, Glaxo-Smith Kline, HMNC, Intra-Cellular therapies, Idorsia, Johnson & Johnson, Lundbeck, Luye Pharma, Medincell, Merck, Mitsubishi Tanabe Pharma, Newron, Novartis, Organon, Orion Corporation, Otsuka, Roche, Rovi, Sage, Sanofi-Aventis, Sunovion, Takeda, Teva, and Viatris, outside the submitted work.

## Declaration of competing interest

The authors declare that they have no known competing financial interests or personal relationships that could have appeared to influence the work reported in this paper.

## The PSY-PGx Consortium

Kristian Kleine Schaars^1,2,3^, Sanah Bedi^1^, Annet Clerx^1^, Janet Strozeski^1^, Allan H. Young^4,5^, Mario Juruena4, Daniel Silman^4^, Urs Heilbronner^6^, Monika Budde^6^, Alba Navarro-Flores^6,7^, Magnus Ingelman-Sundberg^8^, Marin Jukic^8,9^, Martien J. H. Kas^10^, Raj Jagesar^10^, Andra Ciucă^11^, Ramona Moldovan^11,12,13^, Markus M. Nöthen^14^, Per Hoffmann^14^, Carina M. Mathey^14^, Alexandra Philipsen^15^, Laura L. Kilarski^15^, Jonathan Laatsch^15^, Tehila Cohen^16^, Estee Rebibo^16^, Noam Shomron^16^, Erik Van der Eycken^17^, Nigel Olisa^17^, Eduard Vieta^18^, Natalia E. Fares-Otero^18^, Thomas G. Schulze^6,19,20^, Therese van Amelsvoort^21^, Bea Campforts^21^, Emma de Brabander^21^, Moritz J. Rossner^22^, Sven P. Wichert^22^, Roos van Westrhenen^1,2,23,24^

^1^ Outpatient Clinic Pharmacogenetics, Parnassia Psychiatric Institute, Amsterdam, The Netherlands

^2^ Centre for Human Drug Research, Leiden, The Netherlands

^3^ Leiden University Medical Center, Leiden, The Netherlands

^4^ Institute of Psychiatry, Psychology & Neurosciences, King’s College London, United Kingdom

^5^ Division of Psychiatry, Imperial College London, United Kingdom

^6^ Institute of Psychiatric Phenomics and Genomics (IPPG), LMU University Hospital, LMU Munich, Germany

^7^ International Max Planck Research School for Translational Psychiatry (IMPRS-TP), Munich, Germany

^8^ Department of Physiology & Pharmacology, Karolinska Institute, Sweden

^9^ Faculty of Pharmacy, University of Belgrade, Serbia

^10^ Groningen Institute for Evolutionary Life Sciences, Faculty of Science & Engineering, University of Groningen, the Netherlands

^11^ Department of Psychology, Babeş-Bolyai University, Romania

^12^ Division of Evolution and Genomic Sciences, University of Manchester, United Kingdom

^13^ Manchester Centre for Genomic Medicine, Manchester University Hospitals NHS Foundation Trust, United Kingdom

^14^ Institute of Human Genetics, University Hospital of Bonn, Germany

^15^ Department of Psychiatry and Psychotherapy, University of Bonn, Germany

^16^ Gray Faculty of Medical and Health Sciences, Tel Aviv University, Tel Aviv, Israel

^17^ Global Alliance of Mental Illness Advocacy Networks-Europe (GAMIAN-Europe)

^18^ Bipolar and Depressive Disorders Unit, Department of Psychiatry and Psychology, Hospital Clinic, Institute of Neurosciences (UBNeuro), IDIBAPS, CIBERSAM, University of Barcelona, Catalonia, Spain

^19^ Department of Psychiatry and Behavioral Sciences, Norton College of Medicine, SUNY Upstate Medical University, Syracuse, NY, USA

^20^ World Psychiatric Association, Geneva, Switzerland

^21^ Maastricht University, Maastricht, The Netherlands

^22^ Systasy Bioscience GmbH, Germany

^23^ St. John’s National Academy of Health Sciences, Bangalore, India

^24^ Institute of Psychiatry, Psychology & Neuroscience (IoPPN), King's College London, London, United Kingdom
